# Reactivity to Specific Types of Daily Stressors Confer Chronic Disease Risk in Midlife and Later Adulthood

**DOI:** 10.1093/geroni/igaf122.2409

**Published:** 2025-12-31

**Authors:** Kelly Cichy, Dakota Witzel, Robert Stawski

**Affiliations:** Kent State University, Stow, Ohio, United States; South Dakota State University, Brookings, South Dakota, United States; Utah State University, Logan, Utah, United States

## Abstract

Affective responses to stressors (e.g., reactivity) are malleable health risk factors in midlife and later adulthood. Less is known about the specific stressors conferring risk, whether affective responses to stressors confer general health risk, or are specific to certain types of stressors. This study uses data from the Midlife in the United States (MIDUS) study and the National Study of Daily Experiences (NSDE) to examine the prospective effect of differential affective responses to specific types of stressors on chronic disease 7-10 years later and age differences in the stress-chronic disease link. Participants included 1,211 (Mage = 55; SD = 11; range = 34-83; 56% female) who participated in the 8-day NSDE daily diary protocol and waves 2 and 3 of the MIDUS. Respondents reported on the types of daily stressors they experienced (e.g., arguments, work overloads, home overloads, network stressors), their daily affect, and self-reported on diagnosed chronic conditions. Covariate-adjusted possion regressions revealed stressor reactivity to arguments (p = .002), home overloads (p = .06), network stressors (i.e., stressors that happen to a close friend or relative; p = .002), and other stressors (p = .001) are associated with increased chronic conditions 7-10 years later. There was no evidence of linear or nonlinear age differences in any of the reactivity effects, suggesting age invariance. Findings provide insights into how reactivity to specific stressor types increases prospective risk for chronic conditions approximately 7-10 years later, suggesting stressor-specific reactivity is a potential intervention target with relevance in midlife to later adulthood.

